# Impact of Wind Speed on Response of Diffusion-Type Radon-Thoron Detectors to Thoron

**DOI:** 10.3390/ijerph17093178

**Published:** 2020-05-02

**Authors:** Yasutaka Omori, Yuki Tamakuma, Eka Djatnika Nugraha, Takahito Suzuki, Miki Arian Saputra, Masahiro Hosoda, Shinji Tokonami

**Affiliations:** 1 Department of Radiation Physics and Chemistry, Fukushima Medical University, 1 Hikarigaoka, Fukushima 960-1295, Japan; 2 Institute of Radiation Emergency Medicine, Hirosaki University, 66-1 Hon-cho, Hirosaki, Aomori 036-8564, Japan; tamakuma@hirosaki-u.ac.jp (Y.T.); m_hosoda@hirosaki-u.ac.jp (M.H.); tokonami@hirosaki-u.ac.jp (S.T.); 3 Graduate School of Health Sciences, Hirosaki University, 66-1 Hon-cho, Hirosaki, Aomori 036-8564, Japan; h20gg701@hirosaki-u.ac.jp (E.D.N.); h18gg206@hirosaki-u.ac.jp (M.A.S.); 4 Fuji Electric Co., Ltd., 1 Fujimachi, Hino, Tokyo 191-8502, Japan; suzuki-takahito@fujielectric.com

**Keywords:** advection, air exchange, Darcy flow, diffusion-type detector, laminar flow, non-Darcy flow, porous medium, radon, thoron, turbulent flow

## Abstract

Air exchange through a porous medium depends partly on a pressure gradient induced in it, i.e., air-flow conditions of the outer air. Consequently, response of diffusion-type detectors to radon and thoron may vary with air-flow conditions surrounding the detectors. This effect may be significant for thoron measurement because thoron has a shorter half-life than radon. The present study examined response of diffusion-type detectors (RADUETs and one AlphaGUARD) to thoron with respect to wind speed using a thoron calibration chamber. Response of RADUETs to thoron increased with wind speed. Response of the AlphaGUARD increased with wind speed, but it became constant at a high wind speed. Different response trends to thoron between the RADUETs and the AlphaGUARD could be qualitatively explained by flow states induced by the pressure gradient in the filter or the sponge of these detectors. For RADUETs, laminar (Darcy) flow was induced in the sponge in the examined wind speed range, which meant that thoron entry into the detector increased with wind speed. For the AlphaGUARD, laminar flow was induced in the filter in the low wind speed range, whereas flow was changed to turbulent (non-Darcy) flow at a high wind speed for which thoron entry into the detector did not depend on wind speed.

## 1. Introduction

Gaseous radon isotopes, ^222^Rn (radon) and ^220^Rn (thoron), in the air are regarded as major naturally existing sources of radiation exposure to the public [1,2]. According to estimates by the United Nations Scientific Committee on the Effects of Atomic Radiation [1], annual effective doses from internal exposure to radon and thoron are 1.2 mSv and 0.1 mSv, respectively. Although major contributors to the internal dose are their decay products, measurements of radon and thoron concentrations are effective in clarifying quantitative relationships between mother radionuclides and decay products in the living environment for use in regulation purposes (e.g., [3,4,5,6,7,8,9]).

The present study focuses on thoron measurement, which is of significance in two points of radiological protection. The first is to assess the internal dose from thoron. Although the dose from thoron is low, it can become a dominant (non-negligible) component for occupants in certain types of dwellings (e.g., cave dwellings, clay dwellings) and dwelling locations (e.g., on monazite-rich land), and thoron concentration should be measured along with radon concentration (e.g., [10,11,12,13,14,15]). The second point is to contribute to reliable measurement of radon concentration. Radon detectors without a function for alpha-energy discrimination have some response to thoron (e.g., [16,17]). If such detectors are used in an environment with both radon and thoron, such as what is generally seen in a natural environment, thoron is measured as “radon” (namely, thoron interferes with the radon measurement). To minimize thoron interference, radon concentration is corrected using thoron concentration, which is simultaneously measured (e.g., by using twin-cup radon and thoron detectors).

Diffusion-type radon and thoron detectors have been used widely for long-term measurements of radon and thoron concentrations to assess inhalation dose (e.g., [18,19,20]). Diffusive entry (i.e., natural air exchange) is employed to sample the air inside the detection volume of the detectors. Air inlets of the detectors are covered with filters or sponges to prevent ambient aerosols including decay products of radon and thoron from infiltrating into the detection volume. Properties of filters or sponges affect the air exchange rate through them. Previous studies formulated the air exchange rate (*γ*) as follows [18,21,22]:

(1) $$\gamma =\frac{D_{p}A}{dV}$$

where D_p_ is diffusion coefficient in a porous medium such as a filter or a sponge (hereinafter filter/sponge), *d* is thickness of the medium, A is area of the air inlet(s), and V is volume of the detector. The diffusion coefficient is constrained by air temperature and porosity and tortuosity of the medium. Changes in the air exchange rate affect the response of the detectors to radon and thoron. Especially, the effect on response would be directly significant for thoron measurement, because thoron has a shorter half-life (55.6 s) than radon has (3.8 d) and thoron entry through a porous medium into the detectors depends largely on the air exchange rate. Indirectly, radon measurement also would be affected because the radon concentration is sometimes corrected by using thoron concentration to minimize thoron interference on the radon measurement. Thus, it is quite important to clarify factors changing the air exchange rate of diffusion-type detectors.

Previous studies have indicated the change in the air exchange rate associated with the diffusion process in a porous medium. Fleischer et al. [23] reported increased permeability of radon through a membrane filter with increase of the membrane temperature. Similar temperature dependence was obtained for polyethylene polymer foils [24]. Sorimachi et al. [25] reported decreased response of a diffusion-type detector to thoron with increased relative humidity. In addition to these studies, it was recently proposed that the air exchange process was controlled by advection as well as diffusion. Omori et al. [26] found changes in the response of a diffusion-type detector to thoron according to the presence and the absence of forced ventilation of the air surrounding the detector. These authors pointed out the possible induction of advective flow through the filter of the detector caused by the forced air ventilation, and they modified Equation (1) to express the air exchange rate, which is linked to the response to thoron, as follows:

(2) $$\gamma =\frac{A}{V}\left(\frac{D_{p}}{d}+u\right)$$

where *u* is air velocity induced by the pressure gradient in the filter. However, the potential effect of the induced advective flow on the change in the air exchange rate remains unclear. This is because the experiment was conducted in the environment, which showed low thoron concentrations of 10–100 Bq m^−3^ with diurnal fluctuations and, consequently, the distribution of the estimated response to thoron was wide [26]. Moreover, only one detector was used for the experiment under the condition with and without the air ventilation. It has not been clarified whether the change in the air exchange rate with respect to wind speed depends on characteristics of detectors.

The present study aims at examining changes in the response of two diffusion-type detectors to thoron (linked to the air exchange rate) against a wide range of wind speeds in more detail than the previous study did [26]. For exposure of the detectors to thoron, a thoron calibration chamber was used to control thoron concentration stably at the orders of 1000–10,000 Bq m^−3^ and also to control wind speed.

## 2. Materials and Methods

### 2.1. Detectors

**Figure 1:**
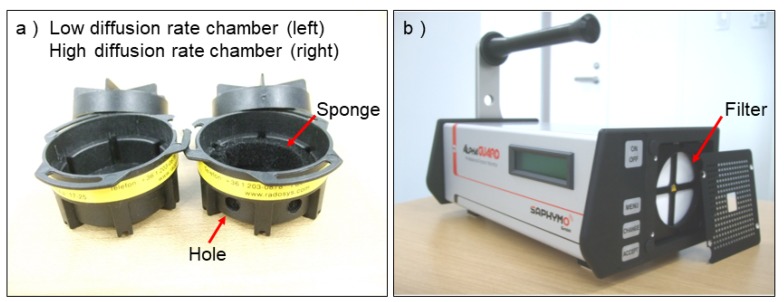
Views of the detectors examined in the present study: RADUET (**a**) and AlphaGUARD (**b**).

Diffusion-type detectors examined in the present study were the solid state nuclear track detectors enclosed in diffusion chambers, marketed as RADUETs (Radosys, Ltd., Budapest, Hungary), and a pulse-ionization chamber, marketed as the AlphaGUARD PQ2000Pro (Saphymo GmbH, Frankfurt, Germany; hereinafter the AlphaGUARD PQ2000Pro is just called the AlphaGUARD) (Figure 1). The former are passive-type radon-thoron discriminative detectors, whereas the latter is an active radon detector that is able to run in the diffusion mode. The AlphaGUARD is typically used to measure radon concentration, but it has a sensitivity to thoron (e.g., [17,26,27,28,29,30,31]) because it does not have a function to discriminate between radon and thoron. In the present study, the AlphaGUARD was used to examine thoron entry into the detector diffusion chamber, which is also useful to clarify thoron interference for radon measurement (Figure 1).

Each RADUET consists of two diffusion chambers with different air exchange rates and a solid-state nuclear track detector CR-39 (BARYOTRAK, Nagase Landauer, Ltd., Ibaraki, Japan) enclosed in each chamber [19,25,32]. The two diffusion chambers are made of electro-conductive plastic with an inner volume of 3.0 × 10^−5^ m^3^. The low diffusion rate chamber detects mainly radon, since there is a diffusion barrier against thoron. On the other hand, the high diffusion rate chamber detects both radon and thoron due to the configuration of the chamber, which has six evenly spaced holes in the cylindrical side wall. An electro-conductive sponge was put on the inner side wall to cover the holes, which prevented ambient aerosols including decay products of radon and thoron from infiltrating into the chamber. For this chamber, radon and thoron gases are introduced into the chamber by natural air exchange through the electro-conductive sponge. The air exchange rates of the low and high diffusion rate chambers (0.71 h^−1^ vs. 10 h^−1^, respectively) differ by two orders of magnitude [25,32], which enables the RADUETs to discriminate radon and thoron without alpha-ray spectrometry. In the present study, only the high diffusion rate chamber of the RADUET was examined (hereinafter RADUET refers to the device using only the high diffusion rate chamber). After exposure of the RADUETs to thoron was completed, they were processed together with control RADUETs at Fukushima Medical University (Japan) to obtain track densities formed on CR-39s. To enlarge the formed tracks, the CR-39s were etched in a 6M NaOH solution at 60 °C for 24 h. The formed tracks were counted at 100× magnification with an upright microscope (ECLIPSE E100, Nikon Corporation, Tokyo, Japan) combined with image processing software, and, consequently, track densities were obtained. The details on processing CR-39 are given elsewhere [12,13].

The AlphaGUARD is a pulse-ionization chamber with a diffusion chamber with an effective volume of 5.6 × 10^−4^ m^3^. It can run in the diffusion or the flow mode according to the measurement purposes. The air inlet of the diffusion chamber is circular with a 6.5 cm diameter, and radon and thoron gases are introduced into the chamber by natural air exchange through a glass-fiber filter covering the inlet when running in the diffusion mode. According to the manufacturer, the glass-fiber filter has a surface density of 70 g m^−2^, a thickness of 0.35 mm, and an average retention capacity of 0.6 μm. Although the AlphaGUARD is a radon detector, it detects thoron at 0.05–0.10 of the actual thoron concentration [17,26,30,31]. That is, when thoron concentration in the air increases from 0 to 100 Bq m^−3^, the value indicated by the AlphaGUARD in the diffusion mode increases by 5–10 Bq m^−3^. The AlphaGUARD examined in the present study was calibrated using a radon calibration chamber established in the National Institutes for Quantum and Radiological Science and Technology (Japan).

### 2.2. Experiment

**Figure 2:**
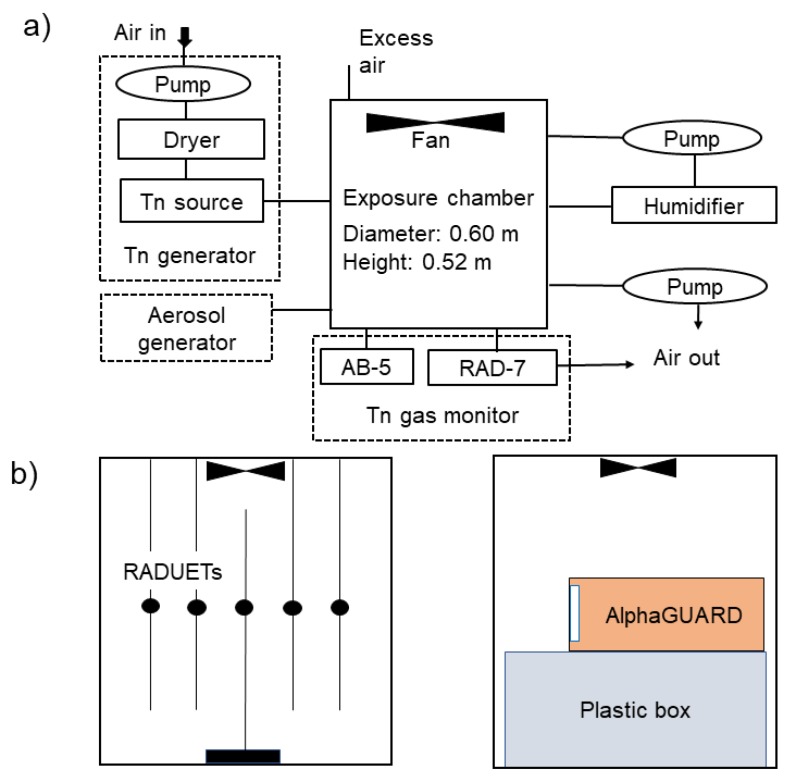
Schematic representations of the thoron calibration chamber system (**a**) and experimental setup for exposures of the RADUETs and AlphaGUARD to thoron (**b**). Panel (**a**) is modified from Pornnumpa et al. [33].

Experiments were conducted by using a thoron calibration chamber established in Hirosaki University (Japan). The thoron calibration chamber system consists of a thoron concentration controller, thoron gas monitors, and a stainless-steel exposure chamber with an inner volume of 1.5 × 10^−1^ m^3^ (dimensions: 0.60 m diameter and 0.52 m height) (Figure 2a; [33]). Commercial lantern mantles were used as the thoron source and stacked together in a cylindrical column. The thoron concentration was controlled by changing the absolute humidity in the air, which passes through the thoron source [34]. The thoron gas generated from the source was injected into the exposure chamber and mixed well by an internal direct current (DC) fan (Model 9WF1224H101, Sanyo Denki Co., Ltd., Tokyo, Japan) installed at the top of the chamber to homogenize the thoron concentration. The thoron concentrations were monitored by alpha-ray spectrometry every hour by a radon-thoron discriminative monitor (RAD7, DURRIDGE Company, Inc., Billerica, MA, USA). During exposures of the detectors to thoron, a grab sampling technique using a scintillation cell (300A, Pylon Electronics Inc., Mississauga, ON, Canada) with a portable radon monitor (AB-5, Pylon Electronics Inc.) was also applied to correct the thoron concentrations measured by the RAD7 [35].

The detectors were set at 26 cm from the top and the bottom of the thoron calibration chamber (Figure 2b). For exposure of the RADUETs, five detectors were hung from the top. Thoron concentrations and exposure times were taken as 12000–16000 Bq m^−3^ and 70–80 h, respectively, and, consequently, time-integrated thoron concentrations were 1000–1300 kBq m^−3^ h to reduce uncertainty of track counting with the microscope. After the exposure was completed, the CR-39s of the sampled RADUETs were processed together with those of the control RADUETs to derive net track densities due to thoron exposure. For exposure of the AlphaGUARD, it was put on top of a plastic box to set the center of the detector inlet at a 26 cm height. The AlphaGUARD was set to the diffusion mode with a measurement cycle of 1 h. Thoron concentrations and exposure times were taken as 900–13,000 Bq m^−3^ and 1–2 d, respectively. For the analysis, successive 12–21 hourly data were used because it took 5–10 h for thoron concentration to become stable in the thoron calibration chamber. Before starting the exposure, radon concentrations in the room where the thoron calibration chamber was located were measured. The results were used as background concentrations to evaluate net values due to the exposure of the AlphaGUARD to thoron.

The exposures of the RADUETs and AlphaGUARD to thoron were conducted at four levels of rotational speeds of the DC fan. The rotational speed was set as very low, low, reference, and high levels. The reference level was the setting for the usual calibration purpose. The lower two levels and the reference level of the rotational speed were controlled by changing the voltage applied to the original DC fan, whereas the highest level was controlled by using another DC fan (Model 9HV1248P1G001, Sanyo Denki Co., Ltd., Tokyo, Japan). Wind speed in the thoron calibration chamber, without the RADUETs or the AlphaGUARD, was measured by using a handheld anemometer. That is, the wind measurement could not be conducted simultaneously with exposure of the detectors to thoron because of the limited space of the small thoron calibration chamber. The non-directional anemometer sensor together with a palm-size data recorder (Model 6542, KANOMAX Japan Incorporated, Osaka, Japan) was put at the 26 cm height in the thoron calibration chamber. Wind speed was measured both at the center and 5 cm from the chamber wall at the four DC-fan rotational speed levels. In each measurement, wind speed was recorded every 5 s for about 1 min. The results are summarized in Table 1.

### 2.3. Analysis

The present study examined changes in response of the RADUETs and the AlphaGUARD to thoron with respect to wind speed in the thoron calibration chamber. Response to thoron was defined as values indicated by the RADUETs or AlphaGUARD divided by thoron concentration in the air surrounding the detectors. The value indicated by each RADUET represented density of tracks formed on CR-39 and that indicated by the AlphaGUARD represented “radon” concentration. Based on these definitions, responses to thoron for the RADUETs (*R*_R_) and AlphaGUARD (*R*_A_) were formulated as follows:

(3) $$R_{R}=\frac{N_{\mathrm{ex}}-N_{\mathrm{bg}}}{C_{\mathrm{Tn},\;\mathrm{out}}\times T}$$

(4) $$R_{A}=\frac{C_{\mathrm{ex}}-C_{\mathrm{bg}}}{C_{\mathrm{Tn},\;\mathrm{out}}}$$

where *C*_Tn, out_ is thoron concentration in the thoron calibration chamber, *N*_ex_ is track density after exposure to thoron is completed, *N*_bg_ is background track density obtained from the control detectors, *T* is exposure time, *C*_ex_ is concentration indicated by the AlphaGUARD during exposure to thoron, and *C*_bg_ is background radon concentration. The response of the RADUETs to thoron shown by Equation (3) can be regarded as a conversion coefficient for exposure to thoron for a CR-39 of the high diffusion rate chamber of the RADUET, and the response of the AlphaGUARD shown in Equation (4) can be regarded as a thoron entry rate described in previous studies (e.g., [17,26,30,31]).

The response to thoron for the detectors can be linked to their air exchange rate. Temporal variation of thoron concentration in a diffusion chamber is formulated as a derivative form with respect to time *t*:

(5) $$\frac{dC_{\mathrm{Tn},\;\mathrm{in}}}{dt}=-\lambda C_{\mathrm{Tn},\;\mathrm{in}}+\gamma \left(C_{\mathrm{Tn},\;\mathrm{out}}-C_{\mathrm{Tn},\;\mathrm{in}}\right)$$

where *C*_Tn, in_ and *C*_Tn, out_ are thoron concentrations in the diffusion chamber of the detector and thoron calibration chamber, respectively, and *λ* is the decay constant (44.9 h^−1^) of thoron [31,32]. When enough time has passed for the thoron concentration to reach equilibrium, the ratio of thoron concentrations in the diffusion chamber to the thoron calibration chamber is a function of the air exchange rate and the decay constant of thoron, and it is formulated as:

(6) $$\frac{C_{\mathrm{Tn},\;\mathrm{in}}}{C_{\mathrm{Tn},\;\mathrm{out}}}=\frac{\gamma }{\lambda +\gamma }.$$

The ratio can be related to the response of the detectors to thoron and, therefore, the air exchange rate.

## 3. Results

**Figure 3:**
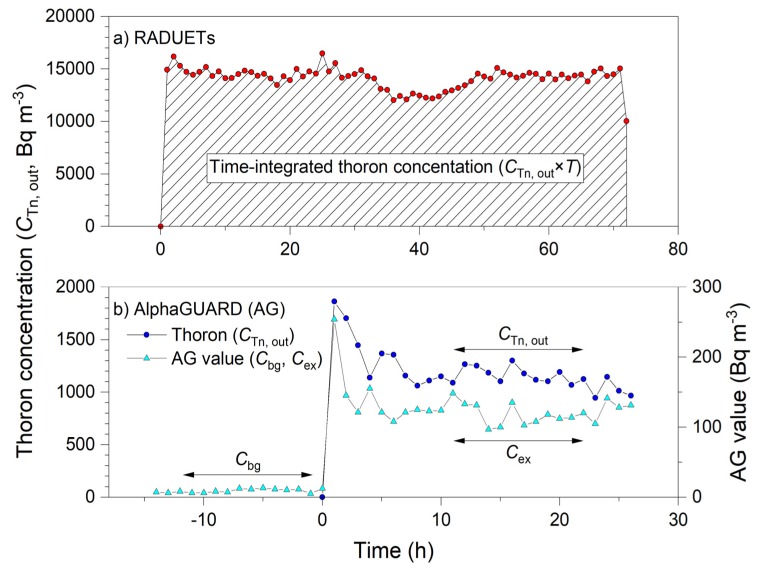
Examples of temporal variations in thoron concentration in the thoron calibration chamber during exposures of the RADUETs (**a**) and the AlphaGUARD (**b**) to thoron. In (**b**), values indicated by the AlphaGUARD are also shown. Meanings of symbols are the same as those in Equations (3) and (4).

Figure 3 shows typical temporal variations in thoron concentration (*C*_Tn, out_) in the thoron calibration chamber during the exposures of the RADUETs and the AlphaGUARD to thoron. In Figure 3b, the *C*_ex_ and the *C*_bg_ values indicated by the AlphaGUARD are presented together with the thoron concentrations. Time-integrated thoron concentration (*C*_Tn, out_ × *T*) depicted in Figure 3a was used to evaluate the response of the RADUETs to thoron expressed in Equation (3). In addition, averages of *C*_ex_, *C*_bg_, and *C*_Tn, out_ depicted in Figure 3b were used to evaluate the response of the AlphaGUARD to thoron expressed in Equation (4).

**Figure 4:**
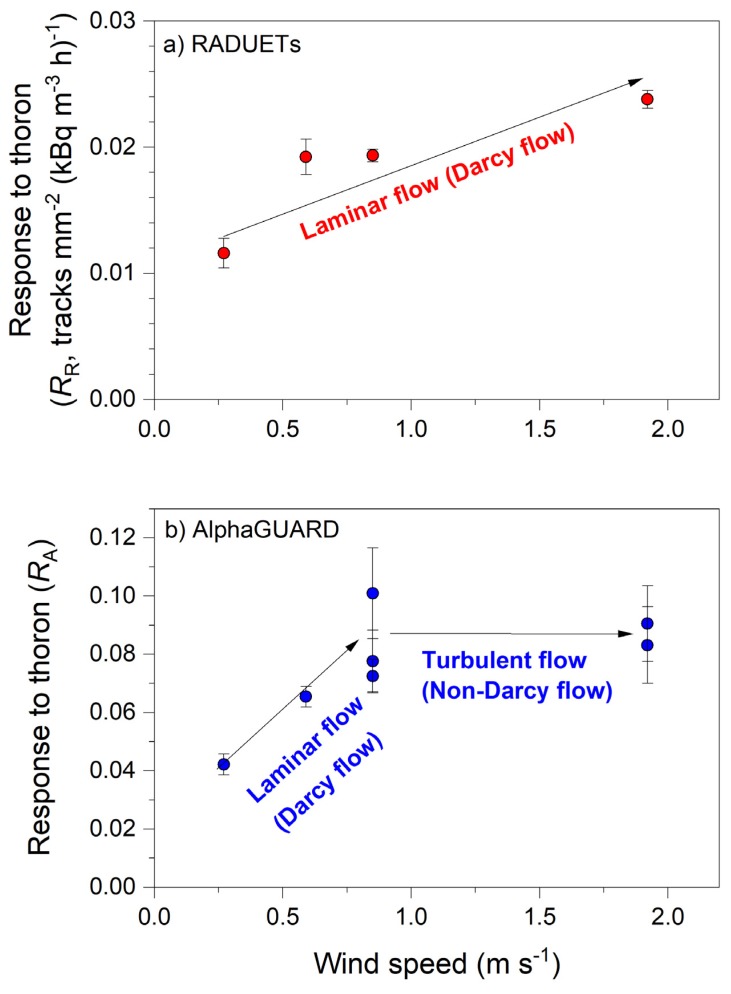
Scatter plots of responses of the RADUETs (**a**) and the AlphaGUARD (**b**) to thoron with respect to wind speed in the thoron calibration chamber. Bars on datapoints represent ranges of one standard deviation.

Table 2 shows the results of exposure of the RADUETs and the AlphaGUARD to thoron under variable wind speeds in the thoron calibration chamber. The relationships between wind speed and the responses of the detectors to thoron are also shown in Figure 4. It is noted again that wind speed was measured in the thoron calibration chamber without setting the RADUETs or the AlphaGUARD in it. It is clearly seen from Figure 4 that the responses to thoron depended on wind speed for both types of detectors, but their trends were different. For the RADUETs, the response to thoron increased with wind speed (Figure 4a). This meant that air exchange was enhanced between the inside and the outside of the RADUETs, and the rate of thoron entry into their diffusion chambers increased with the increase in wind speed. For the AlphaGUARD, the response to thoron increased with wind speed at the very low to reference fan speed levels, which was similar to the trend of the RADUETs. However, it appeared to be constant above the reference fan speed (Figure 4b). In this range, air exchange was insensitive to the change in wind speed of the air surrounding the detector, and the rate of thoron entry into the detector diffusion chamber did not vary. These different trends between the RADUETs and the AlphaGUARD could possibly be attributed to properties of the filter/sponge covering the detector inlets.

## 4. Discussion

The response to thoron (therefore, the air exchange rate) for the RADUETs and the AlphaGUARD varied with wind speed in the thoron calibration chamber. Different trends in the response to thoron with respect to wind speed were seen between these two detectors (Figure 4). Equations (1) and (2) imply that detector geometry (the inlet area divided by the detector volume) affects the air exchange rate, but this geometry value was similar between the RADUETs and the AlphaGUARD (5.7 m^−1^ vs. 6.3 m^−1^, respectively). The occurrence of the different trends was caused by properties of the filter/sponge. Visual observations showed that the sponge covering the inlet of the RADUETs was more porous than the filter covering that of the AlphaGUARD. In fact, the air exchange rate of the RADUETs was 10 h^−1^ [25], 2.5 times higher than that (4 h^−1^) of the AlphaGUARD, which was based on the assumption that the response to thoron was 0.10 of the actual thoron concentration. These findings implied that flow dynamics probably differed between the porous media of the filter and sponge.

**Figure 5:**
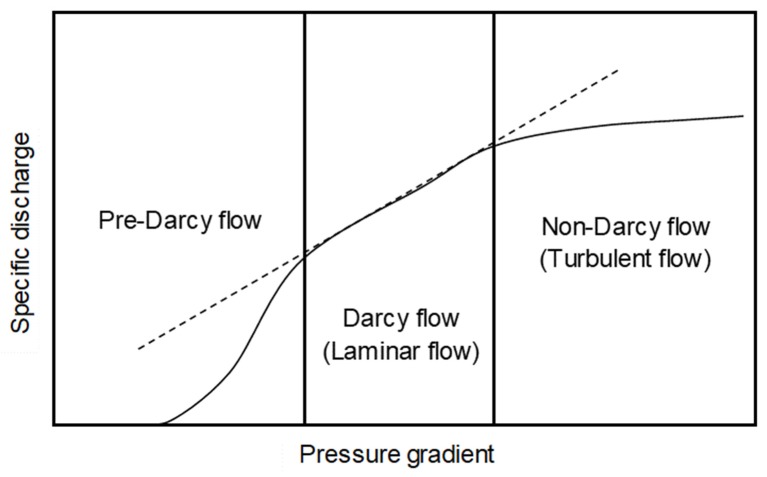
Schematic representation of flow regimes in a porous medium. The broken line follows Darcy’s law. This figure is modified from Basak [37] and Macini et al. [40].

Flow of fluids (including gases) in porous media is classified according to the degree of deviation from Darcy’s law (e.g., [36,37,38,39,40]). Darcy’s law states that the specific discharge is proportional to the pressure gradient for flow through a porous medium. This relationship holds in a certain range of pressure gradient where viscous force is dominant, and laminar (Darcy) flow is maintained. Outside this certain pressure gradient range, specific discharge deviates from that predicted by Darcy’s law. In particular, in a higher pressure gradient range, inertial force becomes dominant and flow changes from laminar to turbulent (non-Darcy) flow. In this situation, the change of the pressure gradient has less impact on specific discharge compared to the impact in the case of laminar flow. Figure 5 shows a schematic representation of flow regimes in a porous medium.

The different trends of the responses to thoron between the RADUETs and the AlphaGUARD can be qualitatively explained in flow regimes in a porous medium. Similar to the representations in Figure 5, specific discharge and pressure gradient are related to the response of the detectors to thoron and the wind field surrounding them, respectively. If wind speed is higher, pressure loss and, therefore, pressure gradient would be higher due to the presence of the filter or sponge. For the RADUETs, the response to thoron increased with wind speed (Figure 4a), which implies that thoron entry into the detectors was constrained by laminar (Darcy) flow induced in the sponge in the examined wind speed range. On the other hand, the response to thoron for the AlphaGUARD increased with wind speed, but it became constant at higher wind speed (Figure 4b). These different trends at lower and higher wind speeds imply that flow regimes for thoron entry into the detector changed from laminar flow to turbulent (non-Darcy) flow in the examined wind speed range. Visual observations showed that the filter of the AlphaGUARD was denser than the sponge in the RADUETs. The denser filter would have a significant impact on the pressure loss caused by the wind field surrounding the detectors. The present study suggests that flow regimes in the filter or the sponge covering the inlet of the detectors play an important role in the changes of the response to thoron with respect to wind field.

Wind direction toward the air inlet(s) of the detectors may affect thoron entry into them. In the present study, because of the limited space of the small thoron calibration chamber, the wind measurement could not be conducted simultaneously with exposure of the detectors to thoron, thus actual wind speed and direction were not determined. Regarding thoron entry into the RADUETs, the influence of wind direction is considered to be small because the six air inlets are spaced equally in the cylindrical side wall of the RADUETs (Figure 1a). However, regarding the AlphaGUARD, wind direction may influence thoron entry into the detector because the air inlet exists only on one side (Figure 1b). The present study showed that the response of the AlphaGUARD to thoron was from 0.04 ± 0.00 (0.042 ± 0.004) to 0.10 ± 0.02 (0.101 ± 0.016) in the examined wind speed range in the thoron calibration chamber (Table 2). The obtained values were comparable to those (0.05–0.10) reported in previous studies (e.g., [17,26,30,31]). Omori et al. [26] reported that the distribution (0.00–0.30; median, 0.06) of the response to thoron in natural air ventilation (wind speed: about 0.03 m s^−1^) was wider than that (0.05–0.23; median, 0.11) in forced air ventilation (wind speed: 0.12 m s^−1^) in the underfloor-space air of a Japanese dwelling because of exposure to low thoron concentration (10–100 Bq m^−3^) with diurnal fluctuations. Unlike that study [26], the present study used the thoron calibration chamber to realize and maintain a high thoron concentration (1000–10,000 Bq m^−3^; Figure 3 and Table 2), which led to lowered uncertainty of the experimental results. There also appears to be a discrepancy in the response to thoron per wind speed between the present study and Omori et al. [26]. The present study evaluated the response as 0.07–0.10 at wind speed of 0.85 m s^−1^, whereas Omori et al. [26] obtained 0.11 at wind speed of 0.13 m s^−1^. This is possibly because, in the present study, wind measurement in the thoron calibration chamber was made without setting the AlphaGUARD in it and, therefore, the actual wind speed during the exposure was expected to be lower than 0.85 m s^−1^. Another possible reason is the different wind fields between these two studies. The wind flowed against the air inlet of the AlphaGUARD in Omori et al. [26], whereas the wind was not necessarily against but rather was circulated by the DC fan in the thoron calibration chamber in the present study. Either or both of these factors might cause the discrepancy.

The findings in the present study provide insights into measurement of thoron using diffusion-type thoron detectors. The present study revealed that the response of the detectors (in particular, the RADUETs) to thoron varied depending on wind speed. This finding does not indicate that the conversion coefficient from track density formed on CR-39 to time-integrated thoron concentration is stable in the environment with a widely variable wind field. It is expected that different conversion coefficients should be given between indoor and outdoor environments and maybe even between indoor rooms with different ventilation rates of the inner air. Use of the single conversion coefficient value causes increased uncertainty in thoron evaluation, which partly leads to increased uncertainty of the radon evaluation corrected by thoron as described above. Therefore, it is necessary to clarify the distribution of the conversion coefficient in the real (natural) living environment.

The present study also provides insights into calibration and intercomparison processes for diffusion-type thoron detectors. Thoron detectors are calibrated in the atmosphere with a known thoron concentration, which is produced, for example, in thoron calibration chambers (e.g., [33,41,42,43,44]). Intercomparison exercises are also important for quality assurance and quality control of thoron detectors (e.g., [44,45,46,47]). An international intercomparison exercise on diffusion-type thoron detectors reported that various thoron concentrations presented by detectors provided by the exercise participants and those relative to the reference thoron concentration were distributed widely from 0.2 to 1.5 [46]. Occurrence of the difference would be natural in part if the participants’ detectors were calibrated under a wind field that was different from that in the thoron calibration chamber of the intercomparison exercise host. That is, the conversion coefficients can differ even for the same detectors exposed to thoron at the same concentration level if calibration conditions used for calibration of the detectors are not the same. Although various factors can be considered, different specifications of thoron calibration facilities among participants’ laboratories can be raised as a potential factor for the cause of the difference.

Some studies reported differences in thoron concentration obtained from simultaneous measurements in environments (dwellings and subsurface workplace) using different diffusion-type thoron detectors (e.g., [48,49]). The above discussion suggested that variable conversion coefficients depending on wind field and different specifications of thoron calibration facilities used for calibration of the detectors may cause part of the reported differences.

## 5. Conclusions

The present study reported variations in responses of two diffusion-type radon and thoron detectors, RADUETs and AlphaGUARD, to thoron under variable wind fields in the thoron calibration chamber. The response of RADUETs to thoron increased with wind speed, which implied that advection in the state of laminar (Darcy) flow caused thoron entry into the detector. In contrast, the response of the AlphaGUARD increased with wind speed, but it became constant at high wind speed. This different trend implied that flow regimes for thoron entry into the detector changed from laminar flow to turbulent (non-Darcy) flow. The finding that the responses of the detectors to thoron varied depending on wind field should provide insights into improving measurement of thoron and processes of quality assurance and quality control for diffusion-type thoron detectors.

## Figures and Tables

**Table 1:** Wind speed in the thoron calibration chamber at four rotational speed levels of direct current (DC) fans.

Rotational Speed Level	Wind Speed (m s^−1^)		
	Center ^1^	Near Wall ^1^	Average ^2^
Very low	0.33 ± 0.01	0.20 ± 0.02	0.27
Low	0.75 ± 0.02	0.44 ± 0.03	0.59
Reference	0.85 ± 0.05	0.85 ± 0.05	0.85
High	1.84 ± 0.05	2.00 ± 0.06	1.92

^1^ Average value ± one standard deviation obtained from the repeated measurements. ^2^ Wind speed averaged at the center and near wall.

**Table 2:** Results of exposure of RADUETs and AlphaGUARD to thoron under variable wind speeds in the thoron calibration chamber.

**RADUETs**					
**Wind Speed (m s^−1^)**	**Exposure Time (*T*, h)**	**Time-Integrated Thoron Concentration (*C*_Tn, out_ × *T*, kBq m^−3^ h)**	**Net Track Density (*N*_ex_–*N*_bg_, tracks mm^−2^) ^1^**		**Response to Thoron (*R*_R_, tracks mm^−2^ (kBq m^−3^ h)^−1^)**
0.27	71	1082	12.6 ± 1.3		0.012 ± 0.001
0.59	82	1281	24.6 ± 1.8		0.019 ± 0.001
0.85	72	1016	19.7 ± 0.5		0.019 ± 0.001
1.92	87	1007	24.0 ± 0.7		0.024 ± 0.001
**AlphaGUARD**					
**Wind Speed (m s^−1^)**	**Exposure Time (*T*, h)**	**Thoron Concentration (*C*_Tn, out_, Bq m^−3^) ^2^**	**AlphaGUARD Value (Bq m^−3^) ^2^**		**Response to Thoron (*R*_A_)**
			***C*** **_ex_**	***C*** **_bg_**	
0.27	21	12752 ± 359	549 ± 42	11 ± 3	0.042 ± 0.004
0.59	17	12294 ± 323	816 ± 37	11 ± 3	0.065 ± 0.003
0.85	16	932 ± 80	98 ± 12	4 ± 2	0.101 ± 0.016
0.85	17	1049 ± 65	89 ± 9	8 ± 3	0.078 ± 0.011
0.85	20	12254 ± 680	900 ± 50	11 ± 3	0.073 ± 0.006
1.92	12	1165 ± 76	115 ± 13	9 ± 3	0.091 ± 0.013
1.92	15	1014 ± 117	94 ± 9	10 ± 3	0.083 ± 0.013

^1^ Average value ± one standard deviation obtained from five detectors. ^2^ Average value ± one standard deviation obtained from 12 to 21 datapoints in successive time series.

## Abbreviations

*A*	Area of the air inlet(s) of the detector (RADUETs or AlphaGUARD)
*C*_bg_	Concentration indicated by AlphaGUARD before starting exposure to thoron
*C*_ex_	Concentration indicated by AlphaGUARD during exposure to thoron
*C*_Tn, in_	Thoron concentration in diffusion chamber of the detector (RADUETs or AlphaGUARD)
*C*_Tn, out_	Thoron concentration in a thoron calibration chamber
*d*	Thickness of a porous medium (filter or sponge)
*D*_p_	Diffusion coefficient in a porous medium (filter or sponge)
*N*_bg_	Background track density on CR-39
*N*_ex_	Track density on CR-39 formed after exposure of RADUETs to thoron is completed
*R*_A_	Responses to thoron for AlphaGUARD
*R*_R_	Responses to thoron for RADUETs
*t*	Time
*T*	Time for exposure of RADUETs to thoron
*u*	Air velocity induced by pressure gradient in a porous medium
*V*	Volume of the detector (RADUETs or AlphaGUARD)
*γ*	Air exchange rate of the detector (RADUETs or AlphaGUARD)
*λ*	Decay constant of thoron
